# A Review on Visible Light Active Perovskite-Based Photocatalysts

**DOI:** 10.3390/molecules191219995

**Published:** 2014-12-01

**Authors:** Pushkar Kanhere, Zhong Chen

**Affiliations:** 1 Energy Research Institute @ NTU, 1 CleanTech Loop, Clean Tech One, Singapore 637141, Singapore; 2 School of Materials Science and Engineering, Nanyang Technological University, 50 Nanyang Avenue, Singapore 639798, Singapore

**Keywords:** perovskite, photocatalysis, visible light active, water splitting, doping

## Abstract

Perovskite-based photocatalysts are of significant interest in the field of photocatalysis. To date, several perovskite material systems have been developed and their applications in visible light photocatalysis studied. This article provides a review of the visible light (λ > 400 nm) active perovskite-based photocatalyst systems. The materials systems are classified by the B site cations and their crystal structure, optical properties, electronic structure, and photocatalytic performance are reviewed in detail. Titanates, tantalates, niobates, vanadates, and ferrites form important photocatalysts which show promise in visible light-driven photoreactions. Along with simple perovskite (ABO_3_) structures, development of double/complex perovskites that are active under visible light is also reviewed. Various strategies employed for enhancing the photocatalytic performance have been discussed, emphasizing the specific advantages and challenges offered by perovskite-based photocatalysts. This review provides a broad overview of the perovskite photocatalysts, summarizing the current state of the work and offering useful insights for their future development.

## 1. Introduction

Photocatalysis has long been studied for clean energy and environmental applications. Over the past two decades, the number of applications based on photocatalysis has increased sharply, while a wide range of materials systems have been developed [1,2,3,4]. Photocatalysis has been of particular interest in the production of hydrogen from water using solar energy [5]. Further, conversion of CO_2_ to hydrocarbons (fuels) is also of significant interest, as it is a solution to reduce CO_2_ emissions across the globe [6,7]. Apart from the clean energy generation, photocatalysis has several promising applications in the environmental field. Some of the applications include degradation of volatile organic compounds (VOC) for water treatment [8], germicide and antimicrobial action [9,10,11], de-coloration of industrial dyes [12,13,14], nitrogen fixation in agriculture [15], and removal of NO_x_/SO_x_ air pollutants [16,17,18,19]. These applications have driven the development of variety of materials systems which are suitable for specific applications. Although TiO_2_-based materials are the most studied for photocatalytic applications, ternary and other complex oxide systems have been increasingly explored as photocatalysts. Among the various classes of materials studied, perovskites-based photocatalysts have unique photophysical properties and offer distinct advantages. 

**Figure 1:**
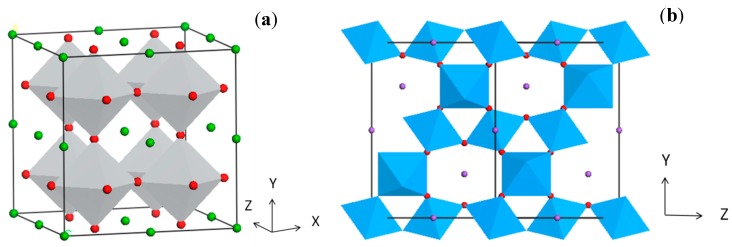
Crystal structure of simple Perovskite, (**a**) BaTiO_3_ and (**b**) double perovskite Na_2_Ta_2_O_6_ (red: oxygen, green and purple: A site cation, grey and blue: BO_6_ octahedra).

Perovskites are the class of compounds presenting the general formula ABO_3_. Generally, in this crystal structure, the A site is occupied by the larger cation, while the B site is occupied by the smaller cation. Perovskites are one of the most important families of materials exhibiting properties suitable for numerous technological applications [20]. Perovskite compounds such as PbZrO_3_, BaTiO_3_, PbTiO_3_ are most commonly used piezoelectric compounds [21]. BiFeO_3_ thin films show multiferroic behavior [22], while compounds such as SrTiO_3_ have shown excellent photocatalytic properties [23,24]. The origin of such properties lies in the crystal structure of perovskites. The perovskite crystal structure has corner connected BO_6_ octahedra and 12 oxygen coordinated A cations, located in between the eight BO_6_ octahedra (Figure 1). The perfect structure of the octahedral connection results in a cubic lattice. However, depending on the ionic radii and electronegativity of the A and B site cations, tilting of the octahedra takes place, which gives rise to lower symmetry structures. As seen from the crystal structure, B site cations are strongly bonded with the oxygen (or any other anion) while, A site cations have relatively weaker interactions with oxygen. Depending on the type of the cations occupying the lattice sites, these interactions could be altered to yield the different perovskite crystal geometries. 

For example, different degrees of tilting of the octahedra give rise to different crystal fields, which result in different electronic and optical properties. The degrees of tilting may affect the band structure, electron and hole transport properties, photoluminescence, and dielectric behavior [25,26]. From the point of view of photocatalysis, perovskite structures may offer significant advantages over the corresponding binary oxides for several reasons. Firstly, perovskites could offer favorable band edge potentials which allow various photoinduced reactions. For example, as compared to the binary oxides, several perovskites have sufficiently cathodic conduction band (CB) energies for hydrogen evolution. Secondly, A and B site cations in the lattice give a broader scope to design and alter the band structure as well as other photophysical properties. In the case of double perovskites such as A_2_B_2_O_6_, stoichiometric occupation of two cations at the B site is known to be beneficial for visible light photocatalysis. Thirdly, some studies have shown that it is possible to combine the effects such as ferroelectricity or piezoelectricity with the photocatalytic effect to benefit the photocatalytic activity.

Perovskite photocatalysts have been studied to a great extent because of their promise for being visible light active. A review of the present work on the visible light driven perovskite photocatalyts is essential to provide a broad overview and possible future directions. Shi *et al.* reported a general review of perovskite photocatalysts active under UV and visible light [27]. The current review article is focused on visible light active perovskite compounds. We emphasize the strategies used to develop or enhance the visible light absorption and subsequent photocatalytic activities. Further, we attempt to shed some light on the underlying principles specific to the perovskite crystal structure which play an important role in the photocatalytic activity, suggesting potential areas in the field where further work is needed. The first section of the article discusses the mechanism and thermodynamics of some of the most important photocatalytic reactions, while the later section reviews the material systems in detail. In the current review, perovskites are broadly divided into simple (ABO_3_ type) perovskites and complex perovskites (double, layered, *etc.*).

## 2. Overview of Photocatalytic Reactions

**Figure 2:**
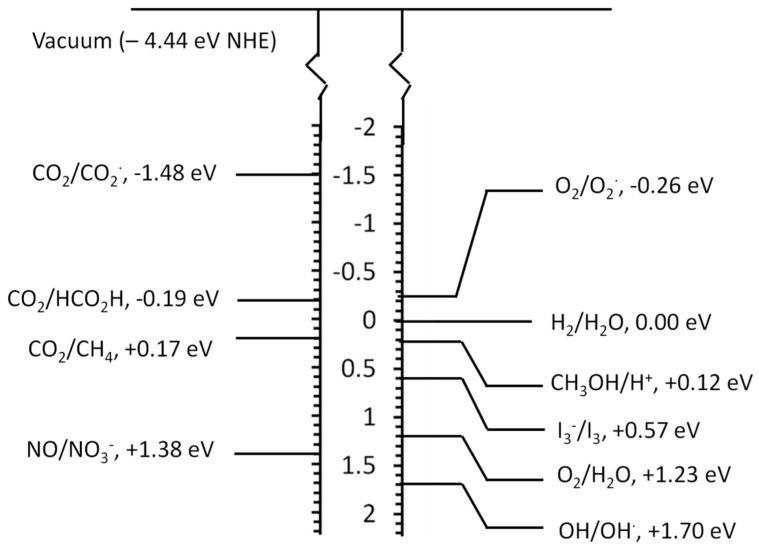
Energy levels of some of the important photocatalytic reactions with respect to NHE at pH = 0 [28].

Photocatalysis is a process that utilizes the energy input from incident radiation and the catalytic properties of the surface of a material to carry out and/or accelerate certain chemical reactions. To date, numerous chemical reactions have been studied, which are potentially useful in energy generation and environmental cleaning applications. Photocatalysis is known to be able to produce thermodynamically uphill reactions, which otherwise need intense energy inputs in terms of high temperature (or pressure). Understanding the mechanism of photocatalytic reactions is critically important to design and develop new photocatalytic materials. In this section, a brief review of mechanism and thermodynamics of most common photocatalytic reactions is presented. Figure 2 shows the reduction and oxidation levels of some of the common photocatalytic reactions with reference to vacuum and the normal hydrogen electrode (NHE). It is noted that these values provide an insight only on the thermodynamic feasibility of the reaction. It is seen that for the reduction reaction, the energy of the (photoexcited) electron should be higher (on the absolute vacuum scale) than the redox level. Therefore the CB potential of the photocatalyst should be located at a higher energy value than the reduction reaction of interest.

### 2.1. Photocatalytic Water Splitting

**Figure 3:**
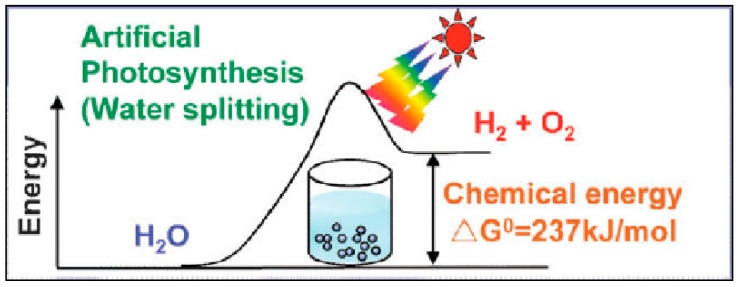
Band diagram and schematics of water splitting reaction over a photocatalyst surface [30].

One of the most studied reactions is the direct splitting of water into hydrogen and oxygen. Figure 3 shows the schematics of the water splitting reaction according to the 4-photon model [29]. 

In the water splitting reaction, upon the radiation of photon with suitable wavelength, photoexcited pairs of electrons and holes are generated within a photocatalyst. Typically, electron-hole separation takes place, due to surface charge or co-catalyst loading. Direct oxidation of water molecules, chemisorbed on the surface of the photocatalyst (or co-catalyst) occurs, by the interaction of water molecule and hole in the valence band (VB) of the photocatalyst. This reaction results in liberation of an oxygen molecule and 4-protons. The protons then migrate to the sites of photoexcited electrons to form hydrogen molecules (Equations (1)–(3)).

2H_2_O → 2H_2_ + O_2_ ∆H = + 234 kJ/mol (1)

2H_2_O → 4H^+^ + O_2_ (2)

4H^+^ + 4e^−^ → 2H_2_ (3)

Evolution of hydrogen and oxygen using sunlight is considered as one of the most promising ways to generate hydrogen as a clean and renewable fuel. Like the water molecule, other molecules also undergo decomposition by the process of photocatalysis.

### 2.2. Photooxidation of Organic Molecules

**Figure 4:**
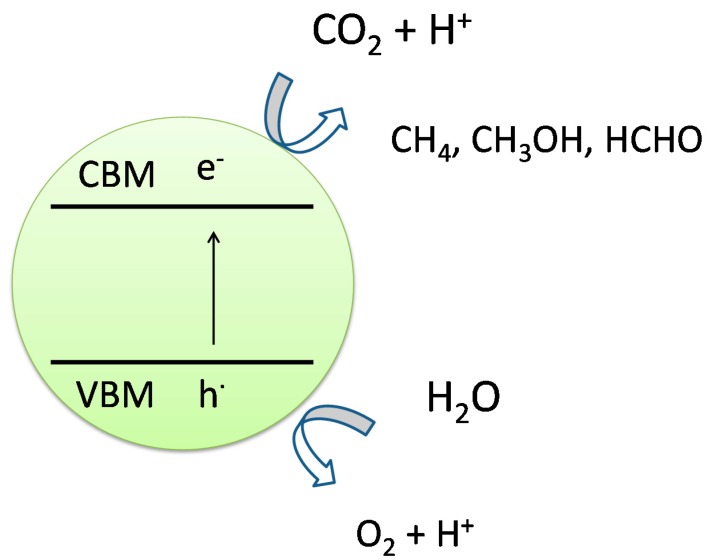
Band diagram and schematics of degradation of organic compounds over a photocatalyst surface [12,33].

Several organic compounds undergo photooxidation reactions, where a direct oxidation via photogenerated holes occurs or an indirect oxidation via hydroxyl ions takes place [31]. The degradation of organic molecules also takes place by reactive oxygen species (Figure 4). Organic dyes, aliphatics and aromatic hydrocarbons, and organic acids can be mineralized to CO_2_ and H_2_O by photocatalytic processes. Like organic compounds, hydroxyl ions and reactive oxygen species (ROS) are known to inactivate microorganisms by degrading their cell walls [10,32]. The photocatalytic inactivation of microbes is effective in antimicrobial, antifungal and antiviral applications. A later section reviews certain silver- and bismuth-based perovskites which display particularly efficient antimicrobial action under visible light. 

### 2.3. Photocatalytic Conversion of CO_2_ to Fuels

**Figure 5:**
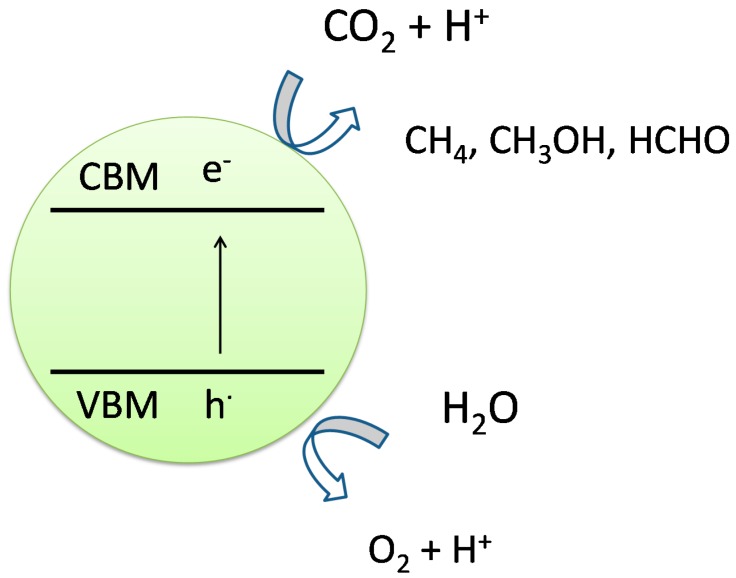
Schematics of CO_2_ photoreduction reaction over a photocatalyst surface [34].

CO_2_, with a standard enthalpy of formation of −393.5 kJ·mol^−1^ at 298 K, is one of the most stable molecules. With appropriate adsorption and photocatalytic processes, reduction of CO_2_ in presence of water could be performed to produce hydrocarbons (Figure 5). Possible chemical reactions of adsorbed CO_2_ and protons are presented by the following equations (Equations (4)–(7)). It could be seen that different number of protons in the reactants, results in different hydrocarbons as products.

Among these reactions, the reaction with eight protons converting CO_2_ to methane is of significant interest. The photocatalytic reduction of CO_2_ in the presence of water is a complex reaction and the photocatalyst must possess enough band potential for proton generation:

CO_2_ + 2H^+^ + 2e^−^ → CO + H_2_O (4)

CO_2_ + 6H^+^ + 6e^−^ → CH_3_OH + H_2_O (5)

CO_2_ + 8H^+^ + 8e^−^ → CH_4_ + H_2_O (6)

2CO_2_ + 12H^+^ + 12e^−^ → C_2_H_5_OH + H_2_O (7)

### 2.4. Photocatalytic Nitrogen Fixation

Like CO_2_ reduction, atmospheric nitrogen could be reduced to ammonia or nitrates by the photocatalytic processes. The mechanism of nitrogen reduction is similar to that of CO_2_, where chemically adsorbed nitrogen molecules react catalytically with protons and form compounds of nitrogen and hydrogen (Equations (8)–(10)):

H_2_O (hv/TiO_2_) → 2H^+^ + _1/2_O_2_ + 2e^−^ (8)

H^+^ + e^−^ → H (9)

N_2_ + H·→ N_2_H (10)

The photocatalytic reduction of nitrogen is extremely useful in nitrogen photofixation processes for agricultural applications [35,36,37]. Although the process of photocatalytic nitrogen fixation is promising, efforts in this area have been severely limited. It is noted that the mechanism of the photocatalytic processes presented above is a simplified understanding, while the photocatalytic processes are complex in nature. 

It is known that a given chemical reaction has a specific photooxidation or photoreduction level (potential) and thus the band potentials of the photocatalyst must satisfy the thermodynamic conditions. Intrinsic properties such as band gap (optical absorption) and band edge potentials determine the thermodynamic feasibility of photoinduced reactions under light irradiation. Apart from the basic conditions, there are several factors which affect the photocatalytic performance of the material system under consideration. Properties such as electron and hole effective mass, exciton lifetime and diffusion length, exciton binding energy affect the electron-hole separation and transport within the lattice. These properties are known to strongly influence the performance (kinetics/efficiency) of the photocatalytic reactions. Defects in the lattice, defect-induced energy states, localization of electrons on specific defect sites could determine the fate of the photoexited electron-hole pair. Finally, the electron transfer across semiconductor-electrolyte interface is significantly affected by surface states, surface band structure (depletion region induced electric field), and band bending. Such electronic properties of materials could be altered to suit specific photocatalytic applications. To date, numerous material systems have been evolved through systematic efforts of understanding and improving the electronic properties of materials. Among these materials perovskites have shown excellent promise for efficient photocatalysis under visible light irradiation, on account of their unique crystal structure and electronic properties. The perovskite crystal structure offers an excellent framework to tune the band gap values to enable visible light absorption and band edge potentials to suit the needs of specific photocatalytic reactions. Further, lattice distortion in perovskite compounds strongly influences the separation of photogenerated charge carriers. The following sections present some groups of materials that have shown visible light activity.

## 3. Simple Perovskites with Visible Light Response 

### 3.1. Titanate Perovskites

**Figure 6:**
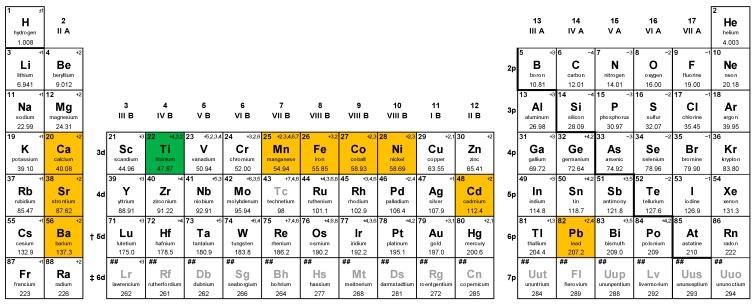
Overview of elements forming perovskite titanates useful for visible light photocatalysis.

Titanate perovskites have been studied for photocatalytic applications for a long time. Most of the titanate perovskites have band gap energy (E_g_) value more than 3.0 eV, however they show excellent photocatalytic properties under UV radiation [1]. Using these titanates as host materials, doping is widely used to alter the optical properties and induce visible light absorption. TiO_2_ (anatase) has a band gap of 3.2–3.4 eV and its CB potential is −0.3 to −0.6 eV above the water reduction level [38]. Certain perovskite titanates have CB energies more negative than TiO_2_, making them more suitable candidates for hydrogen generation. Titanates also offer good photostability and corrosion resistance in aqueous solutions. In this section, a detailed review of MTiO_3_ (M = Sr, Ba, Ca, Mn, Co, Fe, Pb, Cd, Ni) systems is presented. Figure 6 gives an overview of elements that form perovskite titanates. 

#### 3.1.1. SrTiO_3_

SrTiO_3_ is a simple cubic (Pm3m, a = 3.9 Å) perovskite with an indirect band gap of 3.25 eV [39]. When loaded with a co-catalyst such as Rh or NiO_x_, SrTiO_3_ shows stoichiometric water splitting under UV radiation [40] and has been studied extensively for developing visible light water splitting catalysts. Doping of the Ti site with Mn, Ru, Rh, and Ir was of significant interest in early days [41]. It is found that these dopants induce mid-gap states in the band gap allowing visible light absorption [42]. Mn and Ru doping are found useful for O_2_ evolution, while dopants like Ru, Rh, and Ir are suitable for H_2_ evolution [41]. Rh-doped SrTiO_3_ thin films also shows cathodic photocurrent from overall water splitting under visible light, where 7% Rh doped SrTiO_3_ showed 0.18% incident photo-to-electron conversion efficiency (IPEC) under 420 nm irradiation [43]. Using Rh-doped SrTiO_3_ as a H_2_ evolving photocatalyst, various Z scheme systems have been developed. In a significant demonstration, a novel electron mediator [Co(bpy)3]^3+/2+^ was used for Rh-doped SrTiO_3_ with BiVO_4_ photocatalyst. Such a system showed a solar energy conversion efficiency of 0.06% under daylight [44]. Efforts in Z scheme photocatalysis have also been targeted towards eliminating the need for electron mediators by preparing composite photocatalysts. In such systems, electrons from an O_2_ evolving photocatalyst recombine with holes from a H_2_ evolving photocatalyst at the interface of the composite. The quality of the interface and the band alignment of the two semiconductors are important factors for the successful realization of mediator-free type Z schemes.

**Figure 7:**
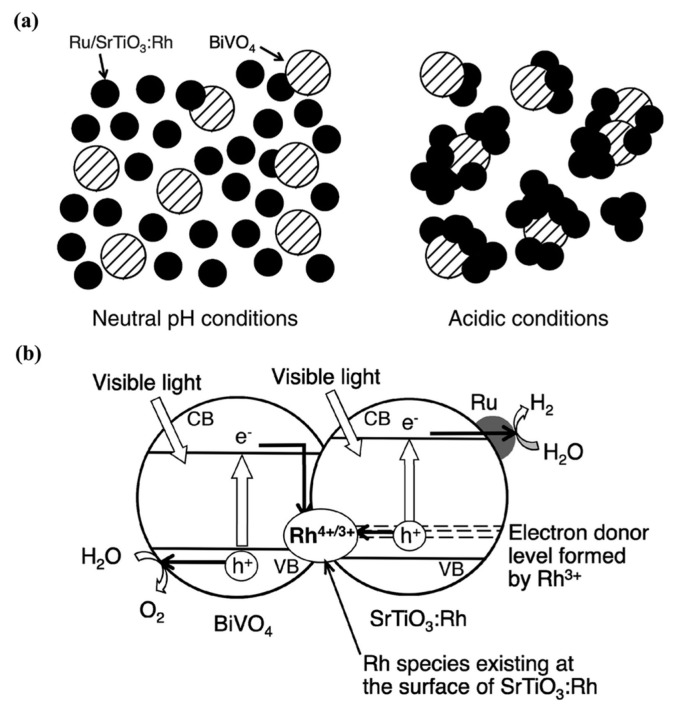
(**a**) Schematic microstructure and (**b**) band diagram of Z scheme photocatalysis using Rh-doped SrTiO_3_ [45].

Rh-doped SrTiO_3_was combined with several O_2_-evolving photocatalysts such as BiVO_4_, AgNbO_3_, Bi_2_MoO_6_, WO_3_, or Cr/Sb-doped TiO_2_ [45]. In those experiments the authors found that agglomeration of the photocatalyst particles occur under acidic conditions, which results in Z scheme photocatalysis. A combination of Rh-doped SrTiO_3_and BiVO_4_ resulted in the best yield [45]. A schematic of microstructure and mechanism of water splitting of an agglomerated Z scheme photocatalyst is shown in Figure 7. In a recent effort, a composite of 1% Rh-doped SrTiO_3_ loaded with 0.7% Ru and BiVO_4_ was successfully prepared. Such a composite showed a stoichiometric water splitting reaction (pH 7) with quantum yield (QY) of 1.6% at 420 nm [23]. These studies successfully establish the feasibility of the “Z scheme” photocatalysis for candidates such as Rh-doped SrTiO_3_ under visible light. 

Further, the water splitting efficiency is dependent on the synthesis method used for Rh-doped SrTiO_3_ [46]. The use of excess Sr in hydrothermal and complex polymerization methods proved useful for improving the apparent yield to 4.2% under 420 nm radiation [46]. Apart from mono-doping, co-doping has been employed in SrTiO_3_ to pursue visible light driven photocatalysis. Co-doping of Sb (1%) and Rh (0.5%) was found useful for visible light photocatalysis and estimated H_2_ and O_2_ evolution rates for 1 m^2^ surface area were 26 mL·h^−1^ and 13 mL·h^−1^, respectively [47]. Further, a composite system was prepared from co-doped La-Cr in SrTiO_3_ and co-doped La-Cr Sr_2_TiO_4_ which showed visible light driven H_2_ evolution (24 µmol·h^−1^·g^−1^) [48]. Composite preparation led to heterojunctions between doped phases and produced a synergistic effect for hydrogen evolution. Further, a solid solution of AgNbO_3_ and SrTiO_3_ was discovered to be a visible light active photocatalyst [49]. (AgNbO_3_)_0.75_(SrTiO_3_)_0.25_ showed promising performance for O_2_ evolution and isopropanol (IPA) degradation under visible light. 

Several efforts have been made to understand and design SrTiO_3_-based photocatalysts. Particularly DFT-based band structure calculations provide useful insights into the electronic structure and its correlation with photocatalytic activity. It is shown that Rh doping in SrTiO_3_ produces band-like states above the valence band maximum (VBM) which are responsible for the visible light absorption. The proximity of dopant-induced states to VBM helps efficient replenishment of electrons and suppresses electron trapping from CB [42]. Theoretical calculations indicate that a TiO_2_-terminated SrTiO_3_ surface with defects such as O and Sr vacancies would alter its electronic structure and induce visible light absorption peaking around 420 nm [50]. Such strategies could be useful in the development of low dimensional materials. Further, theoretical work on doped SrTiO_3_ compounds show that certain dopants such as La strongly lower the effective mass of electrons and holes (near the valence band region), increasing the mobility of the photoexited carrier [51]. Along with the ground state band structure calculations, study of electron and hole masses, defect chemistry, photoexcited transport could be carried out to understand this system in detail. Understanding the excited state properties is useful in further development of new photocatalysts.

#### 3.1.2. BaTiO_3_

Like SrTiO_3_, Rh doping in BaTiO_3_ has been carried out and a quantum yield of 0.5% under 420 nm was reported [52]. Being a hydrogen evolving catalyst, this material has also been used as Z scheme with Pt/WO_3_ for overall water splitting [52]. 

#### 3.1.3. CaTiO_3_

**Figure 8:**
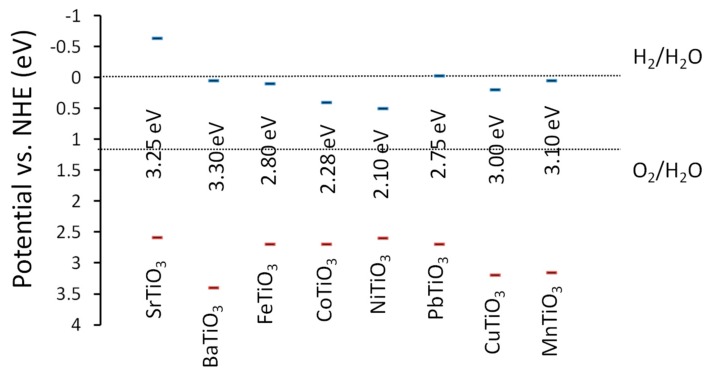
Band edge potentials (*vs*. NHE; pH = 0) of MTiO_3_ system [56].

Calcium titanate is one of the common perovskite minerals with a band gap of 3.6 eV. Cu doping in CaTiO_3_ is widely studied and visible light-driven photocatalytic water decomposition has been reported [53]. Cu doping not only induces visible light absorption, but also enhances hydrogen evolution under UV radiation when NiO_x_ co-catalyst is used. Studies of such doped systems, where dopants enhance the photocatalytic activity host materials are important to design efficient photocatalysts. Detrimental effects of doping on the properties such as electron-hole recombination, electron/hole effective mass, and reduced crystallinity should be studied and reported in detail. These studies are useful to gain insights on the photocatalytic activities of the doped systems. Co-doping of Ag and La at CaTiO_3_ has been done to narrow the band gap and induce visible light absorption [54]. DFT studies also indicate that like SrTiO_3_, TiO_2_-terminated CaTiO_3_ surfaces possess the capability of visible light absorption [55]. Along with alkali titanates, several transition metal titanates show promise for visible light photocatalysis. Figure 8 shows the empirically estimated band diagram of the MTiO_3_ systems with respect to water oxidation and reduction levels.

Alkali metal titanates such as Ba, Ca, and Sr have enough CB potential for hydrogen evolution. However, certain transition metal titanates do not possess the desired CB potential for water reduction, though they have band gap values in the visible region (such as Co, Ni, Fe *etc.*). These materials could be suitable for degradation of organics or other photooxidation reactions. 

#### 3.1.4. CoTiO_3_

CoTiO_3_ has a band gap in the visible region (E_g_ 2.28 eV). Recently this compound has been investigated for photocatalytic O_2_ evolution reaction without any co-catalyst [57]. A yield of 64 μmol·g^−1^·h^−1^ was obtained under visible light. CoTiO_3_ and TiO_2_ composites have been studied for 2-propanol mineralization, however it is worth noting that the band positions of CoTiO_3_ are not suitable for transferring photoexcited charges to TiO_2_, offering no significant advantage [58]. 

#### 3.1.5. NiTiO_3_

NiTiO_3_ has a reported band gap of around 2.16 eV and its light absorption spectra show peaks in visible region corresponding to crystal field splitting [59]. NiTiO_3_ nanorods have been employed for degradation of nitrobenzene under visible light [59]. 

#### 3.1.6. FeTiO_3_

FeTiO_3_ has a band gap of 2.8 eV and thus it absorbs visible light. Composites of FeTiO_3_ and TiO_2_ are studied for the degradation of 2-propenol under visible light. In such composites, TiO_2_ acts as hole capturing phase, thereby separating the electron-hole recombination [60]. 

#### 3.1.7. CdTiO_3_

CdTiO_3_ (E_g_ 2.8 eV) nanofibers were synthesized and studied for the photodegradation of rhodamine 6G (R6G) dye [61]. 

#### 3.1.8. PbTiO_3_

PbTiO_3_ has a band gap of 2.75 eV and has been investigated for visible light photocatalysis. Core-shell particles of nano-TiO_2_ (shell) and micro-PbTiO_3_ (core) were studied for effective charge separation and photocatalytic performance [62]. It is worth noting that PbTiO_3_ is ferroelectric in nature while TiO_2_ is dielectric. It is shown that the ferroelectric behavior helps electron-hole separation at the interface of two particles. 

#### 3.1.9. MnTiO_3_

Further, F doped MnTiO_3_ shows improved separation of charges and degrades rhodamine B under visible light [63]. Some of the promising titanates photocatalysts have been listed in Table 1, as a part of compilation of perovskite systems.

**Table 1:** Compilation of promising photocatalytic systems for hydrogen or oxygen evolution under visible light.

Material System	Irradiation (nm)	Photocatalytic Performance	Experimental Details	Ref.
1% Rh doped SrTiO_3_ (0.5% Pt)	420–800	H_2_ at 48.1 µmol·h^−1^ with sacrificial agent	20% methanol, 50 mg in 50 mL of solution	[64]
Rh: SrTiO_3_: BiVO_4_	>420	Z scheme Water splitting. H_2_ at 128, O_2_ at 61 µmol·h^−1^	4.2% Efficiency, 50 mg 120 mL (FeCl_3_ shuttle)	[46]
Cr-Sb co-doped SrTiO_3_, (0.3% Pt)	>420	H_2_ at 78, O_2_ at 0.9 µmol·h^−1^ with sacrificial agents	in aqueous methanol and AgNO_3_ solution	[65]
MCo_1/3_Nb_2/3_O_3_ (0.2% Pt)	>420	H_2_ at 1.4 µmol·h^−1^ with sacrificial agent	500 mg catalyst in 50 mL methanol, 220 mL water,	[66]
Sr_1-x_NbO_3_(1% Pt)	>420	H_2_ at 44.8 µmol·h^−1^ with sacrificial agent	0.025M oxalic acid, 0.1g catalyst in 200 mL,	[67]
AgNbO_3_-SrTiO_3_	>420	O_2_ at 162 µmol·h^−1^ with sacrificial agent	0.5 g catalyst in 275 mL AgNO_3_ solution,	[49]
LaFeO_3_ (Pt co-catalyst)	400–700	H_2_ at 3315 µmol·h^−1^ with sacrificial agent	H_2_ = 3315, µmol·h^−1^,1 mg in 20 mL of ethanol	[68]
CaTi_1_x_Cu_x_O_3_ (x = 0.02), NiO_x_ co-catalyst	>400	H_2_ at 22.7 µmol·h^−1^ with sacrificial agent	100 mg catalyst in 420 mL methanol solution	[53]
PrFeO_3_, (Pt co-catalyst)	200W Tungsten source	H_2_ at 2847 µmol·h^−1^ with sacrificial agent	1 mg in 20 mL ethanol solution	[69]
Bi doped NaTaO_3_	>400	H_2_ at 59.48 µmol·h^−1^ with sacrificial agent	100 mg catalyst in 210 mL of methanol solution	[70]
GdCrO_3_—Gd_2_Ti_2_O_7_ composite	>420	H_2_ at 246.3 µmol·h^−1^ with sacrificial agent	4.1% apparent quantum efficiency, methanol solution	[71]
CoTiO_3_	>420	O_2_ at 64.6 µmol·h^−1^ with sacrificial agent	100 mg in 100 mL 0.04M AgNO_3_ and La_2_O_3_ solution, 420 nm	[57]

### 3.2. Tantalate Perovskites 

Alkali tantalates are particularly known for efficient overall water splitting reaction under UV irradiation as they possess both VB and CB potentials suitable for water splitting reaction [72,73,74]. To enable visible light photocatalysis, doping of various elements has been studied to achieve visible light activity. 

#### 3.2.1. NaTaO_3_

**Figure 9:**
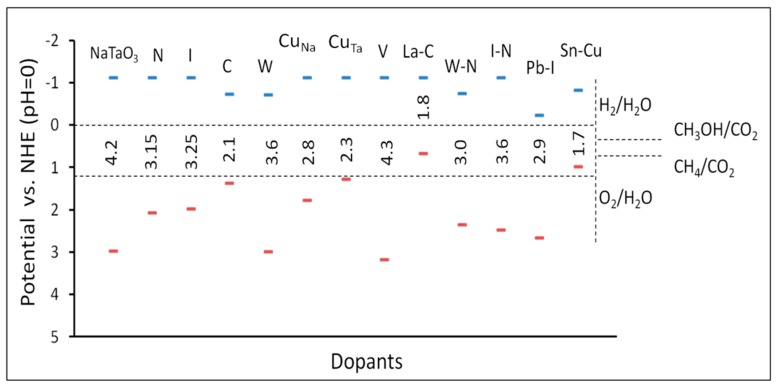
Estimated band gaps and band edge potentials of doped and co-doped NaTaO_3_ systems: DFT study to design novel photocatalyst [85]

Our group reported a detailed study on Bi-doped NaTaO_3_ and showed that the bismuth doping site significantly affects the photocatalytic activity for hydrogen evolution [75,76]. Further, co-doping of La-Co, La-Cr, La-Ir, La-Fe in NaTaO_3_ have shown successful visible light absorption and subsequent hydrogen evolution [77,78,79,80,81]. Co-doping of La-N in NaTaO_3_ has been studied for hydrogen evolution by Zhao *et al.* [82]. These studies have indicated that both anion and cation doping in NaTaO_3_ is useful for visible light photocatalytic applications. Among the doped NaTaO_3_ systems, computational studies on the anionic (N, F, P, Cl, S) doping were reported by Han *et al.* which shows that certain anions like N and P may be useful for visible light absorption [83]. Additionally, doping of magnetic cations such as Mn, Fe, and Co in NaTaO_3_ has also been studied using DFT-PBE [84]. Recently, our group studied DFT calculations of a number of doped NaTaO_3_-based photocatalysts by PBE0 hybrid calculations (Figure 9) [85]. Further, anion doping was also explored in detail using (HSE06) hybrid DFT calculations, where N, P, C, and S doping at O sites were studied. The study also reports the thermodynamics and effect of coupling between N-N, C-S, and P-P on the optical and electronic properties [86]. DFT studies are useful in explaining the properties of existing materials systems and designing new materials. Particularly, use of hybrid functional such as PBE0 or HSE06, is able to accurately define the valence band structure and location of bands or energy states that are crucially important for visible light driven photocatalysis. Hybrid DFT calculations could be useful in predictive modeling, where, band gaps and band edge potential of useful doped photocatalysts are identified. An example of doped tantalate systems is shown in Figure 9.

#### 3.2.2. AgTaO_3_

AgTaO_3_ exhibits similar behavior to alkali tantalates, however, it has a smaller band gap value of 3.4 eV. AgTaO_3_ doped with 30% Nb absorbs visible radiation and shows a stoichiometric overall water splitting reaction under visible light when loaded with NiO co-catalyst [87]. Co-doping of N-H and N-F in AgTaO_3_ has been studied in detail. The study indicates that co-doping not only balances the charges but also improves the carrier mobility. N-F co-doped AgTaO_3_ has an effective band gap value of 2.9 eV and shows H_2_ generation under visible light [88]. 

#### 3.2.3. KTaO_3_

KTaO_3_ (E_g_ 3.6 eV) photocatalysts have been studied for water splitting under UV radiation. However, work on development of visible light driven KTaO_3_ based photocatalysts is limited. 

### 3.3. Vanadium and Niobium Based Perovskites

Similar to tantalum (Ta)-based photocatalysts, Niobium (Nb)-based photocatalysts show good photocatalytic activity under UV irradiation. 

#### 3.3.1. KNbO_3_ and NaNbO_3_

Both KNbO_3_ (E_g_ 3.14 eV) and NaNbO_3_ (E_g_ 3.08 eV) have band gap values in the UV-responsive region, however suitable modifications of the band structure have resulted in visible light photocatalysis [89]. N-doped NaNbO_3_ is a known visible light photocatalyst for the degradation of 2-propanol [90]. Nitrogen doping in KNbO_3_ has been studied for water splitting as well as organic pollutant degradation [91]. First principles calculations predict that co-doping of La and Bi would induce visible light response in NaNbO_3_ [92]. Recent work on ferroelectric perovskites of KNbO_3_-BaNiNbO_3_ shows that the solid solution of these compounds could absorb *six* times more light and shows *fifty* times more photocurrent than others [93]. Although photocatalytic properties are not known, this is an attractive candidate for visible light driven photocatalyst. 

#### 3.3.2. AgNbO_3_

Replacing an A site alkali metal by silver reduces the band gap of the perovskite and AgNbO_3_ has a band gap of around 2.7 eV. Studies have shown that the photocatalytic activity of AgNbO_3_ strongly depends on the shape of the particles: polyhedron-shaped particles are favorable for O_2_ evolution reactions [94]. Further, La doping was found to enhanced the hotocatalytic performance by 12-fold for gaseous 2-propenol degradation [95]. 

#### 3.3.3. AgVO_3_

AgVO_3_ exists in two types of crystal structures, viz. α-AgVO_3_ (E_g_ 2.5 eV) and β-AgVO_3_ (E_g_ 2.3 eV) [96]. Both phases are photocatalytically active. However, β-AgVO_3_ shows better photocatalytic performance than the α-phase. The CB potential of AgVO_3_ is not sufficient for H_2_ evolution, but it is suitable for the degradation of volatile organic compounds (VOCs) and O_2_ evolution. β-AgVO_3_ nanowires show excellent photocatalytic performance in the degradation of Rh B [97]. Composites of AgBr-AgVO_3_ were reported to display respectable efficiency for Rh B degradation [98], while Ag-loaded AgVO_3_ has shown good performance for degradation of bisphenol [99]. 

#### 3.3.4. CuNbO_3_

CuNbO_3_ crystallizes in the monoclinic structure and has a band gap of 2.0 eV. It is an intrinsic p-type semiconductor and has shown 5% efficiency for photon to electron conversion when used as a photocathode. Being a stable material under irradiation, more investigations should be carried out on this material [100]. Tantalum, niobium, and vanadium belong to the same group in the periodic table. Perovskite compounds of these elements show decreasing band gap and CB potential values. This trend is attributed to the 3 d, 4 d and 5 d orbital energies in V, Nb and Ta, respectively.

### 3.4. Ferrite Perovskites

Most of the ferrite perovskites have their native band gaps in the visible region. Hematite and other iron oxide compounds have known shortcomings such as short exciton diffusion length, low electron conductivity, and lower conduction band edge potential [101]. However certain ferrite-based perovskites have shown good photocatalytic activities, circumventing the shortcomings seen in binary iron oxides.

#### 3.4.1. LaFeO_3_

LaFeO_3_ (E_g_ 2.1 eV) has been explored for degradation of pollutants as well as hydrogen evolution under visible light. Sol-gel synthesized LaFeO_3_ loaded with Pt co-catalyst showed high yield of hydrogen evolution (3,315 μmol·h^−1^·g^−1^, in the presence of ethanol) under 400 W tungsten light source [68]. Another study on this phase demonstrates high yield of H_2_ and O_2_ (1290 μmol and 640 μmol after three hours, respectively), without any co-catalyst loading [102]. Further, Thirumalairajan *et al.* showed shape dependent photocatalytic activity of LaFeO_3_ for Rh B dye under visible light (>400 nm) [103]. Doping of Mn in LaFeO_3_ has also been studied and it shows higher photocatalytic activity [104]. Lanthanum ferrite has demonstrated excellent photocatalytic activity under visible irradiation; however, studies on the fundamental photophysical properties are lacking the literature. Understanding the reasons why LaFeO_3_ is a better photocatalyst that Fe_2_O_3_, in terms of comparative electronic properties such as electron-hole separation, mobility, photoexcited lifetimes *etc.* is important for further development in ferrite-based photocatalyst.

#### 3.4.2. BiFeO_3_

**Figure 10:**
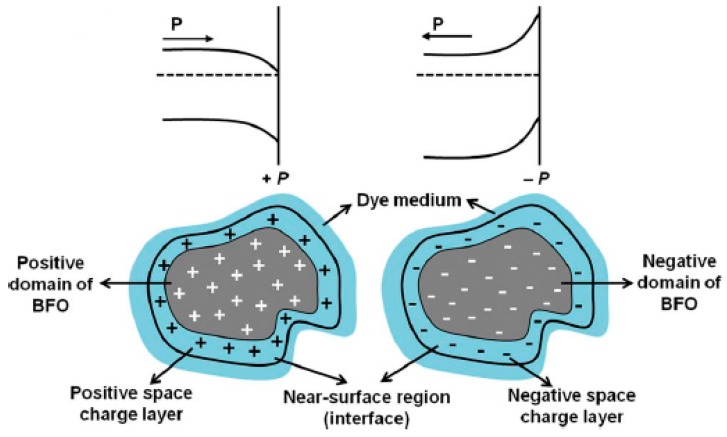
Schematics of changes to band diagram upon polarization of Gd doped BiFeO_3_ due to ferroelectricity [106].

BiFeO_3_ is a known ferroelectric material with a band gap of 2.3 eV. Recent studies have shown that BiFeO_3_ could be used as a visible light photocatalyst [105]. The ferroelectric properties of BiFeO_3_ could be utilized to enhance the electron-hole separation and improve the photocatalytic activity. Figure 10 shows the effect of polarization on the band bending of BiFeO_3_ particles. Band bending affects the separation of electrons and holes in the space charge region and thus affects the photocatalytic activity. Gd-doped BiFeO_3_ show enhanced photocatalytic degradation for rhodamine B degradation attributed to its ferromagnetic behavior [106]. Ca doping in BiFeO_3_ leads to improved performance for photocatalytic degradation of Congo Red dye [107].

In another study, cations such as Y, Mg and Al were doped in BiFeO_3_ and their photocatalytic performance was evaluated by degradation of Rh B under 400 nm radiation. The degradation performance was limited to C/C_0_ of 0.8 within three hours [108]. The study of the effects of ferroelectric behavior on photocatalytic performance is a relatively new topic and it generally shows impressive activity for the degradation of organic pollutants, however, more experimental evidence and understanding are needed to establish a correlation between ferroelectric behavior and photocatalytic activity. Further studies on the stability and toxicity of bismuth-based materials should be carried out for realizing their practical applications. It is further noted that the tilting of octahedra in the perovskite crystal structure significantly affects its electronic properties. Nevertheless, the effect of tilting of octahedra on the photophysical properties such as electron-hole separation, electron transport, delocalization of charges has not been studied in detail. Such studies will prove useful in establishing the importance of perovskites in the field of photocatalysis.

#### 3.4.3. GaFeO_3_

GaFeO_3_ has been reported to show overall water splitting without any co-catalyst loading under visible light (λ > 395 nm) [109]. The authors also reported a yield of 0.10 and 0.04 µmol·h^−1^ under a 450 nm band pass filter. It is worth noting that the catalytic activity decreased due to deactivation of the catalyst. 

#### 3.4.4. YFeO_3_

Among the other ferrites, YFeO_3_ has a band gap value of 2.43 eV and showed photocatalytic activity *four* times that of TiO_2_-P25 under > 400 nm visible light radiation (Rh B degradation) [110] 

#### 3.4.5. PrFeO_3_

PrFeO_3_ was evaluated for hydrogen evolution reaction from ethanol-water mixture and showed a yield of 2847 μmol·g^−1^·h^−1^ under 200W tungsten lamp irradiation [69]. 

#### 3.4.6. AlFeO_3_

Composites of AlFeO_3_ and TiO_2_ are reported to yield the sunlight driven photocatalytic degradation of methyl orange (MO) and eosin dye [111]. Ferrite based perovskites also offer advantage of magnetic recovery of the particles which is useful in practical applications. 

### 3.5. Other Perovskite Systems

Among the other perovskite photocatalysts, compounds of bismuth, cobalt, nickel, and antimony (occupying B sites) have band gap values in the visible region. Pentavalent bismuth perovskites are known to be active photocatalysts under visible light radiation. Perovskites such as LiBiO_3_ (E_g_1.63 eV), NaBiO_3_ (E_g_2.53 eV), KBiO_3_ (E_g_2.04 eV), and AgBiO_3_ (E_g_ 2.5 eV) have all been investigated for degradation of organic pollutants [112]. NaBiO_3_ shows better photocatalytic performance for phenol and methylene blue (MB) degradation as compared to other Bi^5+^ containing perovskites as well as P25-TiO_2_ [112]. NaBiO_3_ is also reported to have higher photocatalytic activity than N-doped TiO_2_ photocatalyst [113]. NaBiO_3_ and BiOCl composites have been studied for enhanced electron-hole separation and subsequent photocatalysis [114]. AgBiO_3_ was shown to be effective in restricting the growth of *Microcystis aeruginosa* under simulated solar light [115]. This study shows that AgBiO_3_ with band gap energy around 2.5 eV could be a potential algaecide under natural light. DFT studies show that NaBiO_3_ has a strong band dispersion arising from Na 3s and O 2p hybridized orbitals, which contributes to the higher photocatalytic activity [112]. LaCoO_3_ has a band gap value of 2.7 eV and oxygen deficient LaCoO_3_ (LaCoO_3-δ_) has been studied for MO degradation (>400 nm) [116]. 

Other compounds such as LaNiO_3_ has been studied for MO degradation using wavelengths greater than 400 nm [117]. Cu doping in LaNiO_3_ has been studied for improved H_2_ evolution [118]. Ilmenite type AgSbO_3_ has an absorption edge onset at 480 nm, and it has been demonstrated for O_2_ evolution under sacrificial agent as well as degradation of MB, RhB, and 4-chlorophenol [119]. Further, solid solution of LaCrO_3_ and Na_0.5_La_0.5_TiO_3_ was developed for hydrogen evolution [120]. Recently, Gd_2_Ti_2_O_7_/GdCrO_3_ composite was reported as photocatalyst p-n junction photocatalyst [71]. The study shows that GdCrO_3_ has a band gap of 2.5 eV and is responsible for the visible light absorption [72]. 

## 4. Complex Perovskite Materials

### 4.1. Double Perovskites

Compounds with general formula A_2_B_2_O_6_ belong to the double perovskites and they have similar crystal structures to simple perovskites. Double perovskites have the basic framework of corner connected BO_6_ octahedra and A cations enclosed within, however, the connectivity of the octahedra may differ from structure to structure. Double perovskites could accommodate different cations at the A or/and B sites, taking a general form AA'BB'O_6_. Accommodation of different cations at the A and B sites alters the photophysical properties of the compound significantly. Among the binary oxides, only a few compounds are known to have band gap values in the visible region (narrow gap), e.g., Fe_2_O_3_, WO_6_, CuO, Bi_2_O_3_. However, these materials suffer from insufficient CB potential for hydrogen evolution. Some of the materials also suffer from poor stability and low mobility of photoexcited charges. On the other hand, many of the binary oxides are known to be efficient photocatalysts, however only activated by UV radiation (wide gap). Complex compounds offer a possibility of combining the elements from ‘narrow gap’ and ‘wide gap’ binary compounds to exploit the properties of both types of oxides, and thus are potentially useful as visible light photocatalysts. The following section reviews photocatalytic properties of double perovskites which are visible light active. 

#### 4.1.1. Sr_2_FeNbO_6_

Sr_2_FeNbO_6_ has a cubic crystal structure and a band gap of 2.06 eV. In its pristine form, it is a known photocatalyst, while 7% Ti doping at Fe site has shown two time enhancement of the hydrogen generation in methanol solution. A total of 28.5 µmol·h^−1^ and 650 µmol·h^−1^ were reported in presence of sacrificial agents and with 0.2% Pt as co-catalyst [121]. W doping in Sr_2_FeNbO_6_ has also been studied and demonstrated enhancement to hydrogen evolution under visible radiation [122]. 

#### 4.1.2. La_2_FeTiO_6_

Hu *et al.* reported higher photocatalytic activity for La_2_FeTiO_6_ than for LaFeO_3_ for degradation of *p*-chlorophenol under visible light irradiation [123]. 

#### 4.1.3. Other Double Perovskites 

Rare earth and bismuth-based double perovskites were studied for visible light photocatalysis. Compounds with general formula Ba_2_RBiO_6_, (R = La, Ce, Pr, Nd, Sm, Eu, Gd, Dy), were prepared and their photocatalytic activity was studied for MB degradation [124]. Authors found rare earth cation dependent photocatalytic performance, where compounds such as Ba_2_EuBiO_6_, Ba_2_SmBiO_6_, and Ba_2_CeBiO_6_ showed high photocatalytic activity. Complex perovskites such as CaCu_3_Ti_4_O_12_ have also been studied for their photocatalytic performance. CaCu_3_Ti_4_O_12_ was found to possess an indirect band gap of 1.27 eV and Pt-loaded CaCu_3_Ti_4_O_12_ shows degradation of MO under radiation greater than 420 nm [125]. Photophysical properties of certain double perovskite compounds have been reported, however, more efforts are needed to investigate photocatalytic properties of these materials. Compounds such as Ba_2_CoWO_6_, Ba_2_NiWO_6_, Sr_2_CoWO_6_ and Sr_2_NiWO_6_ are stable perovskites compounds for O_2_ evolution using sacrificial agents [126]. 

Certain tantalum-based compounds have been studied for degradation of organic compounds. N-doped K_2_Ta_2_O_6_ is known to absorb visible light up to 600 nm and degrade formaldehyde under visible light [127]. Our group demonstrated that Bi doping in Na_2_Ta_2_O_6_ causes visible light absorption and degradation of MB [128]. Although some work has been done with double perovskites as photocatalysts, understanding of their fundamental properties is limited. More work is needed to discover their advantages as visible light photocatalyst and develop novel materials systems.

### 4.2. Mixed Oxides 

Mixture of oxides and nitrides or oxides and sulphides has been developed to engineer the band structure of the oxide photocatalysts suitable for the visible light absorption. Oxynitride and oxysulphide photocatalysts offer distinctive advantages over their doped counterparts. Earlier reports on oxynitrides have revealed that replacing oxygen by nitrogen at lattice sites, in a stoichiometric manner, narrows the band gap of the oxide, by pushing the VBM into the band gap [129]. Such a modification does not produce native point defects, which would otherwise be introduced in the case of N doping. Stoichiometric incorporation of nitrogen also avoids the localized states induced by N doping and reduces the possible electron-hole recombination. Similar composition containing mixtures of sulfur and oxygen *i.e.*, oxysulfides, has been developed for photocatalysts. Mixed oxysulfide perovskites of Sm_2_Ti_2_S_2_O_5_ (E_g_ 2.0 eV) is known for water oxidation and reduction reaction for oxygen and hydrogen evolution, respectively in presence of sacrificial agents under low photon energy wavelengths of 650 nm. The band structure of this phase reveals that the presence of sulphur narrows the band gap and enables visible light absorption [130]. 

Oxynitride compounds such as CaNbO_2_N, SrNbO_2_N, BaNbO_2_N, and LaNbON_2_ belong to the perovskite type crystal structures [131]. Photocatalytic hydrogen evolution has been reported under visible light from methanol solution. SrNbO_2_N (E_g_ 1.8 eV) has been investigated in detail, where the photoelectrode of SrNbO_2_N on a transparent conducting surface shows water oxidation reaction under no external bias [132]. Tantalum counterparts of these compounds were developed and utilized in the Z scheme photocatalysis. Compounds such as CaTaO_2_N and BaTaO_2_N were loaded with Pt co-catalyst and coupled with pt/WO_3_ for Z scheme water splitting [133]. A solid solution of BaTaO_2_N and BaZrO_3_ was formulated for hydrogen and oxygen evolution, which showed improved performance compared with the individual photocatalysts under visible light [134]. LaTiO*_x_*N*_y_* is another perovskite type compound which shows high photocurrent density under visible light [135]. 

Apart from the double perovskites belonging to the general formula AA'BB'O_6_, there are a several other compounds that show crystal structures close to the perovskite type structure, however such compounds are not included in the current review. Theoretically, double perovskites offer a wider scope to design photocatalysts by selecting suitable cations and AA' and BB' sites in the lattice. Work on design and development of double perovskite is currently limited and synthesis and characterization of new materials in this category are needed. 

## 5. Summary and Outlook

A large number of perovskite-based compounds (over 80) have been studied for visible light driven photocatalytic applications. Perovskite structures offer abundant scope in designing novel compounds based on A and B site occupancy, which gives rise to a wide range of materials systems with unique properties. Among these compounds, photocatalysts with pristine band gaps in the visible region such as LaFeO_3_, PrFeO_3_, NaBiO_3_, and AgBiO_3_ (Table 2) show promising photocatalytic performance under visible radiation (>400 nm). Although there are reports on the photocatalytic activity of these compounds, detailed studies on these materials are limited. More efforts are needed to understand the structure-property relations in such ferrites and bismuth based compounds and improve their photocatalytic activity. Among the wide band gap semiconductors, SrTiO_3_- and NaTaO_3_-based photocatalysts are the most investigated systems. The strategy of doping foreign elements in wide band gap photocatalysts is widely used to induce visible light absorption and subsequently to enable photocatalytic activity. Nonetheless, very limited knowledge is available on the adverse effect of dopants on the photophysical properties of the compounds (e.g., if the benefit derived from dopant-induced visible light activity is overweighed by a loss of UV light activity). This should be properly investigated in the future. Appropriate dopants which retain the beneficial properties of the host materials while inducing visible light responses should be identified. Research work on complex perovskites show that these compounds offer distinct advantages as compared with simple perovskites. Designing complex perovskite compounds with suitable elements at A and B sites to yield desired photocatalytic properties is a challenge. We expect computational design would help shorten the selection process. Recent advances in computational tools such as DFT based band structure calculations are highly effective to design and understand novel materials systems.

**Table 2:** Compilation of promising photocatalytic systems for organic compounds degradation under visible light.

Materials System	Band Gap (eV)	Photocatalytic Tests Reported	Ref.
Ga doped BiFeO_3_	2.18–2.50	Enhanced degradation of rhodamine B compared to pristine BiFeO_3_	[106]
LaFeO_3_	2.10	Nanospheres show higher rates of rhodamine B degradation than nanocubes and nanorods	[103]
YFeO_3_	2.43	Rhodamine B degradation rate higher than P25 (>400 nm)	[110]
NaBiO_3_	2.60	Bleaching rate of Methylene Blue higher than N doped TiO_2_. (>400 nm)	[113]
AgSbO_3_	2.58	Eddicient degradation of Rh B. MB, 4-chlorophenol (>420 nm)	[119,136]
AgBiO_3_	2.50	Inhibition of *Microcystis*	[115]

As certain perovskite compounds exhibit ferroelectric, ferromagnetic, or piezoelectric effects, there is a need to understand the correlation between these effects and the photocatalytic activity to a greater depth. Such studies would certainly be helpful in the development of efficient visible light photocatalysts. On a final note, there has been significant progress in the development of visible light perovskites in the past years. This development has laid a good foundation for future work in this area. Further understanding of the crystal and electronic structural factors behind photocatalytic activity is needed for the future development of efficient visible light-driven perovskites.
